# Mainstreaming nature-based solutions through five forms of scaling: Case of the Kiiminkijoki River basin, Finland

**DOI:** 10.1007/s13280-023-01942-0

**Published:** 2023-10-24

**Authors:** Simo Sarkki, Olli Haanpää, Hannu I. Heikkinen, Juha Hiedanpää, Karoliina Kikuchi, Aleksi Räsänen

**Affiliations:** 1 Cultural Anthropology, University of Oulu, PO Box 1000, 90014 Oulu, Finland; 2 Max Weber Centre for Advanced Cultural and Social Studies, Erfurt University, Erfurt, Germany; 3 Natural Resources Institute Finland (Luke), Itäinen Pitkäkatu 4a, 20520 Turku, Finland; 4 Natural Resources Institute Finland (Luke), Paavo Havaksen Tie 3, 90570 Oulu, Finland

**Keywords:** Benefits, Management fixes, Participatory workshops, Policy fixes, Relational values, Scaling-up

## Abstract

Nature-based solutions (NBS) are considered as means to tackle climate change and biodiversity loss while simultaneously enhancing human well-being. Yet, it is still poorly understood how NBS could be mainstreamed. We address this gap by proposing a framework on NBS and employing it in Finland’s Kiiminkijoki River basin through participatory workshops and a questionnaire. We examine socio-environmental challenges and visions, existing and emerging NBS to reach the visions, and ways to scale-up NBS to a river basin level. In the river basin, water quality is the priority challenge, due to its relationships with local culture, climate change, and biodiversity. Our results consider how (1) to ensure the relevance of NBS for local actors, (2) instrumental, intrinsic, and relational value perspectives can be enhanced simultaneously by NBS, and (3) site specific NBS can be mainstreamed (i.e., by scaling up, down, out, in, deep) to the river basin level and beyond.

## Introduction

Current sustainability challenges, including climate change, biodiversity loss and enhancing human well-being, require urgent solutions to balance environmental and social aspects (Rockström et al. 2023). Nature-based solutions (NBS) refer to solutions to the sustainability challenges that are inspired and supported by nature (Maes and Jacobs 2017; Nesshöver et al. 2017; Raymond et al. 2017). NBS have the potential to contribute to mitigating and adapting to climate change, halting biodiversity loss, and ensuring human well-being (Chausson et al. 2020; Seddon et al. 2020). NBS can do so, for example, by reducing anthropogenic carbon emissions (Pan et al. 2023). To address climate change, the implementation of NBS needs to be effective at the global level, but also appropriate for the socio-economic and physical conditions prevailing at the local level (Cong et al. 2023). Regarding biodiversity loss and enhancing human well-being, NBS can reduce existing inequalities and enhance various ecosystem services in the locations where the NBS are implemented (Balzan et al. 2021). Moreover, spatial approaches have been developed to identify and prioritize sites for NBS implementation in a way that enables multiple benefits (Guerrero et al. 2022) and provides the best balance of ecosystem services contributing to human well-being (Longato et al. 2023). A key challenge common for such location-based approaches is to mainstream the NBS for societal transformations to address pressing sustainability challenges though scaling-up.

Societal and cultural change has been considered a necessary feature of the sustainability transformation (see IPBES 2019). Indeed, many long-term modifications in how humans interact with nature are needed, which calls for mainstreaming NBS across society through scaling. The rationale of scaling is that promising, often niche based NBS initiatives, involving new practices and governance approaches need to be duplicated, extended, or used to inform subsequent policy development (Fastenrath et al. 2020). While recent papers have addressed scaling-up NBS (Fastenrath et al. 2020; Cortinovis et al. 2022; Odongo et al. 2022), other forms of scaling have not been yet addressed in connection to NBS.

To conceptualize various forms of scaling, we draw insights from literature on social innovation to enrich understanding on mainstreaming NBS through scaling. Because NBS like social innovations are often quite local, their replication to wider scales has emerged as an important topic of interest. Social innovation literature has identified five forms of scaling: (1) scaling-up (producing changes in laws, norms, policies), (2) scaling-out (geographically replicating or broadening the range or scope of good practices), (3) scaling-down (resource allocation to support implementation), (4) scaling-in (ensuring organizations’ capacity to engage in required good practices required), and (5) scaling-deep (value changes in society) (Westley and Antadze 2010; Moore et al. 2015; Sánchez Rodríguez et al. 2021).

We examine NBS and their scaling through the case of the Kiiminkijoki River basin located in northern Finland. The Kiiminkijoki River drains an almost 4000 km^2^ peatland-dominated basin in rural northern Ostrobothnia, a region characterized by a sparse and aging population, long distances, a changing economic structure and a relatively high level of unemployment. The key challenge in the region is to implement solutions that can help to meet carbon neutrality, enhance biodiversity, and maintain or increase livelihood possibilities.

Our objective is to examine NBS and their potential mainstreaming in the Kiiminkijoki River basin case based on participatory workshops and a questionnaire. Our research questions are:

1. What are the local stakeholders’ perceptions of challenges and visions for the river basin development?
2. How the challenges can be addressed, and visions achieved through NBS?
3. What does it require to mainstream the NBS by scaling (-up, -out, -down, -in, -deep) to the river basin level and beyond?

## Framework to examine nature-based solutions

This article is part to the field of environmental governance studies. Our framework recognizes that NBS can enhance sustainability by providing benefits for nature and people. Yet, achieving sustainability requires societal and cultural change. Therefore, novel operative insight is needed on the values underpinning and undermining necessary societal and cultural change to be mainstreamed by NBS. We approach the challenge of mainstreaming NBS from the analytic perspective of scaling. We emphasize that when scaling NBS, the related needs must be analyzed (i.e., benefits and values for people and nature) and connected to the ways NBS is mainstreamed (i.e., by scaling-up, -out, -down, -in, and -deep environmental management and policy fixes).

NBS have been defined in various ways, and there is no standardized definition (Sowińska-Świerkosz and García 2022). The EU Research Innovation policy agenda defines NBS as *“Solutions that are inspired and supported by nature, which are cost-effective, simultaneously provide environmental, social and economic benefits and help build resilience”* (Dumitru and Wendling 2021). The concept of NBS has been applied commonly in urban contexts (e.g., Almenar et al. 2021; Cortinovis et al. 2022; Pan et al. 2023) and to lesser extent in river basin areas (Liquete et al. 2016; Thorslund et al. 2017; Reaney 2022). For us, NBS are management and policy fixes that enhance mutually beneficial relationships between people and nature and that can be mainstreamed by the five forms of scaling.

The concept of ecosystem services (ES) helps understand and operationalize the co-beneficial relationships between nature and people: ES are benefits people obtain from ecosystems (e.g., MA 2005). Importantly, the concept of nature’s contributions to people (NCP) has recently been proposed as an update to the ES approach. NCP highlights the dynamic aspects of nature through the capacity of ecosystems to remain flexible and maintain good quality of life and recognizes the diverse knowledge systems and perspectives regarding the values of nature (Díaz et al. 2018; Peterson et al. 2018).

Within the ES and NCP literature it is considered that values and benefits for both people and nature can be understood through the intrinsic, instrumental, and relational values of nature (Pereira et al. 2020; IPBES 3 August 2022). In terms of intrinsic values of nature, NBS may contribute to the maintenance and enhancement of ecosystems by leading benefits to their functions (see Comberti et al. 2015). In terms of instrumental values, NBS offer an opportunity for innovation as a cost-effective way of creating a greener, more sustainable, and more competitive economy (Faivre et al. 2017), and provide a set of ecosystem services (Keesstra et al. 2018). In terms of relational values, NBS link to the cultural ways diverse people relate to nature. Relational values are historical, reflecting the co-evolutionary qualities of the relationships between humans and nature, such as care, social bonding, place attachment and spiritual meanings (Pascual et al. 2017; Chan et al. 2018; Mattijssen et al. 2020). Recognizing the diversity of cultural relations to nature by relational values could open new ways to engage with local communities (Buijs et al. 2022). Furthermore, NBS-related science, policy and practice needs to broaden the instrumental ecosystem service lens towards understanding the constitution of ecosystem services as an inclusive, collaborative assemblage with human–nature connections (Welden et al. 2021). Thus, instrumental, intrinsic, and relational values need to be considered as complementary to each other (Chan et al. 2016).

NBS are often considered as environmental management fixes: people shape, engineer, restore, conserve, manage, modify, or create new ecosystems with NBS (e.g., Cohen-Shacham et al. 2016; Potschin et al. 2016; Nesshöver et al. 2017). The idea is that healthy natural and managed ecosystems produce a diverse range of services contributing to human well-being, from storing carbon, controlling floods, and stabilizing shorelines and slopes to providing clean air and water, food, fuel, medicines, and genetic resources (MA 2005).

We consider that management fixes cannot function without also changing and shaping human behavior. Therefore, NBS is a policy concept, including the necessary element of collective decision-making (see Malekpour et al. 2021; Zingraff-Hamed et al. 2021) that places focus on engagement with various stakeholders (Potschin et al. 2016). Management fixes become policy fixes in the context of NBS. Policy fixes include hard policy (e.g., laws and taxes that restrict choices and alter financial incentives) but also soft policy (e.g., educational campaigns, nudges and collaborative arrangements) (e.g., Banerjee et al. 2021). Furthermore, market-based instruments (Jordan et al. 2005) and strategies (Freedman 2013) help solve problems in human-nature relationships by combing hard and soft policy features. Examples of policy fixes linked to NBS are the legal establishment of nature protection areas, incentives and ecological compensations to change peoples’ behavior, regulations for increasing restoration, biodiversity, and climate mitigation plans and practices. Policy fixes are also needed to compensate and cover the costs and burdens of NBS for those people suffering from those approaches (see Giordano et al. 2020; Seddon et al. 2021).

**Fig. 1:**
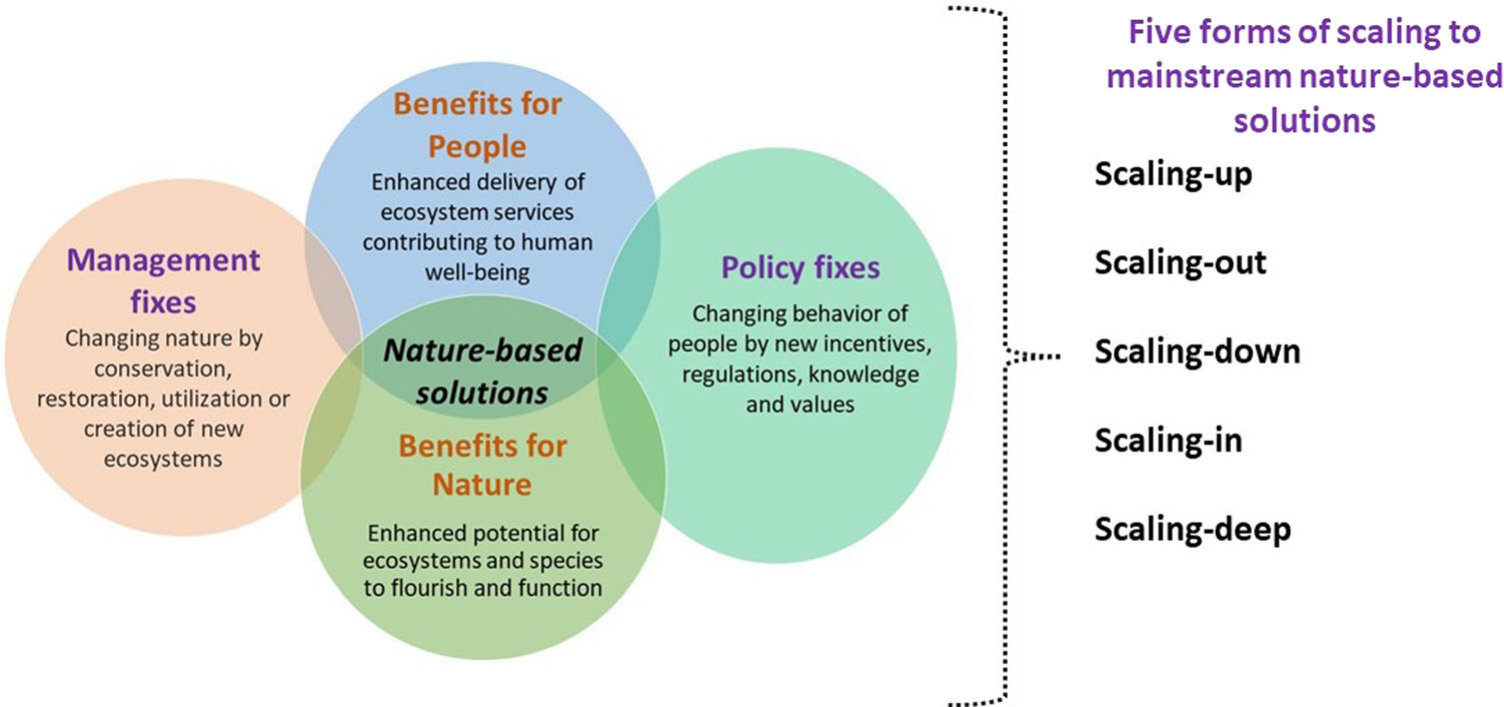
Heuristic framework to understand NBS and ways to mainstream them across society

NBS can be applied at a landscape scale, for example a river basin, which often includes a variety of ecosystems such as forests, peatlands, wetlands, and agricultural areas (Cohen-Shacham et al. 2019). Even if remaining local, it is important to consider how NBS may be scaled-up (Cohen-Shacham et al. 2019; Fastenrath et al. 2020; Cortinovis et al. 2022; Odongo et al. 2022). Scaling-up NBS means mainstreaming and amplifying NBS from highly localized solutions to wider applications (see Fastenrath et al. 2020; Frantzeskaki and McPhearson 2021; Schröter et al. 2022). Apart from scaling-up, other forms of scaling have not been explicitly recognized in NBS literature. Social innovation literature has identified five forms of scaling (Westley and Antadze 2010; Moore et al. 2015; Sánchez Rodríguez et al. 2021), which are also relevant for NBS. Sánchez Rodríguez et al. (2021) identified four forms and directions for scaling social innovation: up (producing changes in laws, policies, institutions or norms), out (geographically replicating or broadening the range or scope of good practices), down (resource allocation to support implementation), and in (ensuring organizations have the capacity to deliver the type and number of good practices required). Moore et al. (2015) identified an additional direction for scaling: deep (impacting on cultural roots by changing relationships, cultural values and beliefs) (Table 1; Fig. 1).

**Table 1:** Five forms of scaling in social innovation literature (Westley and Antadze 2010; Moore et al. 2015; Sánchez Rodríguez et al. 2021) and related examples from NBS literature

Forms of scaling	Definitions from social innovation literature	Examples from NBS literature
Scaling-up	Initiating changes in laws, policies, or norms based on promising lower-level management practices	Fastenrath et al. (2020) *“The underlying rationale is that successful (niche) [NBS] initiatives testing new practices, services or governance approaches, should be duplicated, linked or enlarged and/or brought to higher policy levels.”*
Scaling-out	Geographically replicating or broadening the range or scope of good management practices	Diffusion of NBS from highly localized solutions to wider application to address sustainability challenges (see Frantzeskaki and Mcphearson 2021; Schröter et al. 2022)
Scaling-down	Ensuring necessary means for policy (e.g., incentives, regulations, nudges) to support the implementation of promising practices at local level	To ensure funding for financing ecological restoration (or other kinds of NBS) (see Cohen-Shacham et al. 2016)
Scaling-in	Adjusting the structure, functions, or skills within an organization to allow it to take on the work required to implement the good practices it is trying to promote	Municipal strategy and planning can help embed NBS in longer planning processes in cities (Hawxwell et al. 2019)
Scaling-deep	Long-term change in management and policy that leads to societal change at the level of practices, norms, beliefs and finally also values	Catalyzing societal value change by seeking to achieve biodiversity benefits and positive social impacts together and to increase the overall success of an NBS (Cohen-Shacham et al. 2016)

The five forms of scaling can help to mainstream NBS across society for sustainability transformation. We emphasize that, like the NCP approach, our framework also recognizes *“the central and pervasive role that culture plays in defining all links between people and nature”* (Díaz et al. 2018, p. 270). Figure 1 highlights NBS’ ability to enhance benefits for people and nature, requiring management fixes as concrete modifications of nature by people. However, such management fixes are accompanied by behavioral change of people supported by policy fixes: new incentives, compensations, regulations, nudges, and rules. To be effective, NBS thus necessitate cultural and societal change, at least in locations where specific NBS are implemented. The five forms of scaling then consider how this cultural and societal change can be mainstreamed across society for wider sustainability transformation.

## Material and methods

### The case of the Kiiminkijoki River basin

**Fig. 2:**
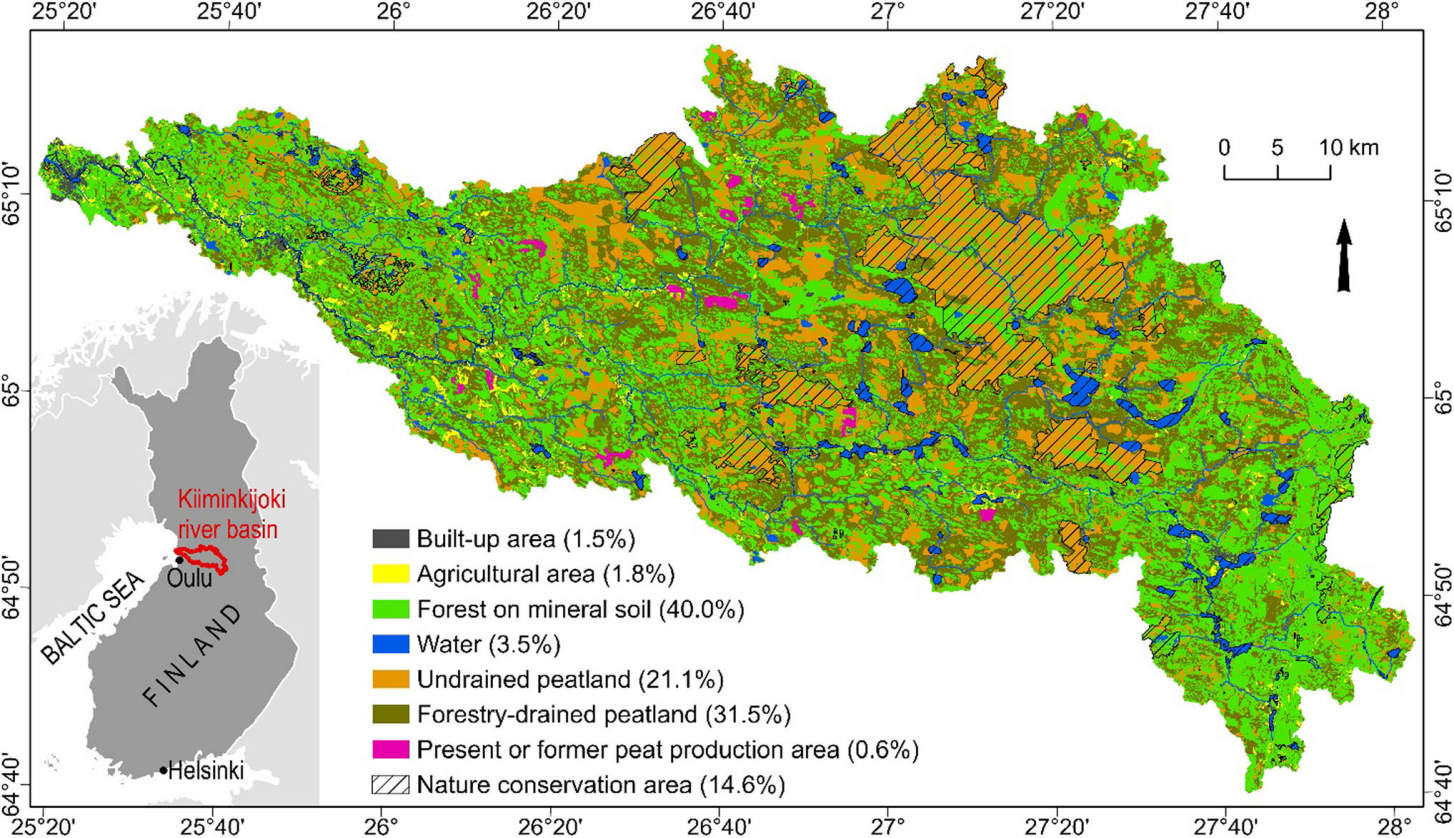
Land use/land cover in the 3824 km^2^ Kiiminkijoki River basin in northern Finland. Data sources: Finnish Environment Institute (Corine Land Cover, peatland drainage status, conservation areas), ELY Centre for North Ostrobothnia (peat production areas), and Finnish Food Authority (land parcel register)

The Kiiminkijoki River drains a 3,824 km^2^ peatland-dominated basin (Fig. 2) in rural northern Ostrobothnia. The economy of the region has been based on primary production, particularly forestry and peat energy production and small-scale agriculture. The region includes tens of thousands of hectares of forestry-drained peatlands, part of which, however, are not economically profitable for forestry use (Juutinen et al. 2020). There are ecological restoration plans targeted for these areas but also e.g., former peat energy production sites and possible spawning grounds for fish. Many of the important plausible restoration sites are located near waterbodies and owned by numerous and heterogenous private landowners.

The ecological water quality is good or even excellent in the upstream, but some tributaries have only satisfactory or even poor quality (Finnish Environment Institute 2023). The water quality problems are due to the diffuse loading of nutrients and suspended solids caused by the drainage of peatlands and intensive land use in parts of the river basin. This has also led to a dramatic reduction of salmon and other migratory fish stocks (Koljonen et al. 2013).

The Kiiminkijoki River basin is a case in a region which is under pressure for the green transition from European policy (Council of Oulu Region 2023). Furthermore, Finland has set a target to become carbon neutral by 2035, and the plan includes the sequestration of carbon in the land use sector, particularly in forest areas on peat and mineral soil (Ministry of the Environment 2023). Regarding biodiversity, the EU restoration law may also amplify pressures to increase the restoration of peatlands and other habitats in the region (Räsänen et al. 2023).

### Empirical materials and analysis methods

We used empirical materials collected in three participatory stakeholder workshops and an online questionnaire (Table 2). The methods employed in workshops included the participatory methods of backcasting future (e.g., Bibri 2018), map-assisted discussion, and reflective discussions about concerns and aspirations linked to the river basin.

**Table 2:** Overview of empirical materials used in the present paper

Empirical material collected from	Participants	Rationale
Half-day kick off stakeholder meeting (April 2022)	Approximately 20 onsite and 10 online participants. The participants were invited by an open invitation that was advertised via email and in a local newspaper. Part of the discussions were held in smaller groups, with 5–10 participants in each group	To obtain an overview of the development needs, interests, and values of the river basin. To gather river basin-scale geographical information and beliefs of the areas that are considered important or in need of restoration
Half-day local workshop for forest-owners and landowners, and an onsite visit to a constructed wetland (October 2022)	Seven participants. We invited the forest owners (*N* = 300) from the vicinity of the sub-catchment to the workshop by email (*N* = 140) and letter (*N* = 160). The workshop was also advertised on social media	To gather more detailed and targeted knowledge of one site, i.e., the constructed wetland. To discuss local-level problems and concerns, and land use solutions to overcome those problems. To provide local information for the river basin-scale workshop (next row)
Full-day participatory future workshop targeting the whole river basin (February 2023)	Twenty-three participants. Participants were personally invited to cover different interests, beliefs, values, and knowledge related to the river basin. The final list of participants included actors from municipalities, regional administration, civil society associations, research organizations, and interest groups. Most of the discussions were held in three groups with 7–8 participants in each group	To discuss desired futures in the river basin and how to reach these goals. To bridge visions, aspirations, and hopes of various organized actors. Discussions were divided into two main sessions. The morning session regarded visioning the desirable futures for the region linked to environmental, economic, governance and cultural developments. In the afternoon session, participants discussed how to achieve those visions
Online questionnaire, launched during spring 2022	Thirty-five respondents representing landowners and organized actors. The link to the questionnaire was distributed via email and social media. 40% of respondents lived in the Kiiminkijoki River basin, 34% own forest in the basin, 50% fish and do recreational activities in the river, 29% own second home in the region, and 26% were born in the area. The age of respondents varied from 30 to 76 with an average of 59 years. Around one quarter of respondents were women	To collect information about the development needs for the river basin and local opinions about the preferred land use measures. The survey included questions related to future objectives that should guide land use in the river basin, ambitions for climate change mitigation, land use measures, governance, and key concerns regarding the future of land use in the region

The main source of materials used in the present paper are the full day participatory workshop and the questionnaire. The workshop agenda started with a morning session on the identification of desirable future visions for the Kiiminkijoki River basin. The visions were discussed especially from the points of view of environment, culture, governance, and economy. After lunch, the same small groups discussed how these visions could be reached. The facilitators asked the groups to consider what needs to happen to achieve the visions, who are responsible for initiating the changes, what are the barriers for the changes, how the barriers can be overcome, and what are the knowledge needs regarding the proposed changes. The workshop ended with recap of group work in plenary.

The online questionnaire included 15 questions on the respondent’s relationship to the Kiiminkijoki River basin, preferred climate change mitigation targets in the region, goals and values that should guide the land use in the river basin, land use options, governance in the river basin, and basic background information. These were closed questions with predetermined options. Open questions covered concerns and hopes regarding the river basin and the land use therein but also promising ongoing projects and practices. In particular, the open future-oriented question on hopes and visions was answered in an extensive way.

Our analysis of the empirical materials integrated deductive and inductive qualitative analysis approaches (see Hsieh and Shannon 2005). We recognized that iterative, interactive, and reflexive qualitative analysis approaches were needed because *“patterns, themes, and categories do not emerge on their own… the role of iteration, not as a repetitive mechanical task but as a deeply reflexive process, is key to sparking insight and developing meaning. Reflexive iteration is at the heart of visiting and revisiting the data and connecting them with emerging insights, progressively leading to refined focus and understandings.”* (Srivastava and Hopwood 2009, p. 77). Consequently, we changed the major research question during the analysis. We first focused our analysis to examine the potential of NBS in nurturing reciprocal relations between nature and people, but then during the iterative and reflexive analysis we shifted the focus from the reciprocity to the mainstreaming of NBS through scaling. Upon the shift, we conducted a literature search and found growing interest, but little examined focus on mainstreaming NBS through the five forms of scaling.

Leading from this finding, we created the framework after the workshop to capture key issues discussed. NBS and their mainstreaming through scaling were chosen as conceptual tools to analyze the empirical materials. The discussed solutions to address the challenges in the river basin are tightly linked to management and policy fixes which aim to restore or create new ecosystems and change human behavior. Mainstreaming through scaling is also an empirically appropriate concept because several promising mainstreaming approaches and ideas were present in our empirical materials. Nevertheless, it was uncertain how they could be mainstreamed at the river basin level to achieve widely shared goals to improve water quality in the river and less consensual goals to improve carbon sequestration and halt biodiversity loss. The empirical materials were re-clustered to answer our refined research questions.

### Conceptual and empirical limitations

We note that we did not use the concept of NBS in any of the workshops or questionnaire. This was because the NBS term is not known by the stakeholders and would probably add confusion. However, the issues discussed in the workshops and considered in the questionnaire are tightly coupled with our conceptualization of NBS as management and policy fixes benefitting both people and nature. These solutions need to be mainstreamed by scaling them to the river basin level to meet the objectives (e.g., improved water quality) that the local stakeholders have.

We acknowledge that the empirical materials used in the present paper may be slightly biased. We received 35 responses to the survey distributed via email and social media. Furthermore, a total of seven forest-owners and landowners participated in the local workshop while the other workshops had more than 20 participants. We do not consider that the sample is statistically representative of the people living in the river basin; instead, the sample indicates the people who are actively interested in the river basin and its development. We consider it likely that the sample represents views that emphasize socio-cultural and environmental values more than economic ones. Particularly, local forest-owners and landowners who emphasize economic values of land and think that there is no need to change current forest and land use management practices are likely underrepresented in our empirical material. Due to the low number of questionnaire respondents, we conducted exploratory data analysis by calculating frequencies to different answer options but did not conduct any statistical tests.

## Results

### Key visions

**Fig. 3:**
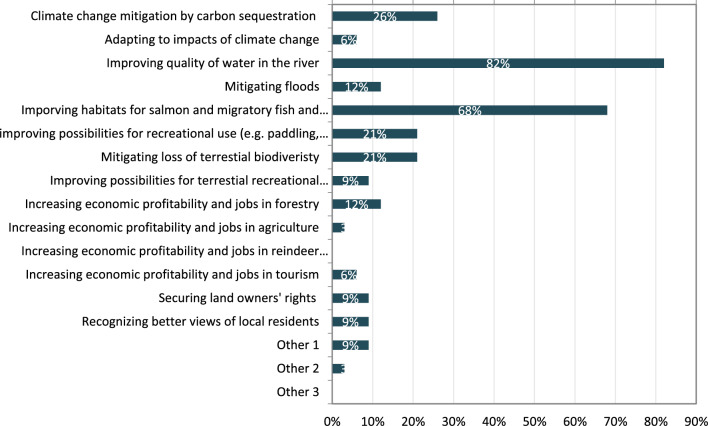
Responses to survey question: what objectives should guide land use in the Kiiminkijoki River basin (*N* = 34). The respondents were able to pick a maximum of three answers

The key finding from the questionnaire (Fig. 3) and the future workshop was that the most consensual future objective is to improve the water quality in the river, which produces also benefits for the people (Table 3). The enhanced water and especially habitat quality could then help salmon return to the river. In addition of providing subsistence for some families, salmon is a symbolically important species linked to the pride of local people in their home region and the river. There is also the possibility to develop tourism around salmon. The enhanced water quality can also help to revive the populations of grayling which is also perceived locally as an important catch for recreational fishing. Furthermore, enhanced water quality in the river is considered to increase the attractiveness of the region for both inhabitants and tourists. Tourism could in the future bring additional income to the region, but it is also recognized that it must be done with caution to not to emerge as a new threat to environmental values.

**Table 3:** Visions for the river basin that participants of the future workshop prioritized in a post-it exercise in three groups

Emerging topic cluster	Group 1	Group 2	Group 3
Water quality	Significant improvements in water quality. Focus on migratory fish Good or excellent ecological state in the river	Improved water quality and engagement of landowners to water protection measures Water quality close to a natural state and all human activities start from respect towards nature Biodiversity enhanced in the river and in the river basin	Water quality is almost as good as in a river in a natural state
Salmon and migratory fish	Strong and stable populations of migratory fish in the river. Local people in the river basin enjoy nature in diverse ways	Salmon and trout spawn in the river	
Nature-based tourism	Top destination for fishing tourism and for paddling in the region		Developing nature-based tourism for example by establishing national park The river is top fishing destination in the region from sea to upstream
Management fixes	Applying extensive water protection measures in forestry	Limited nutrient flows to the river	Rethinking of ditches in the whole river basin and directing water flows to natural channels and wetlands
Well-being in the region	A living countryside where people and livelihoods flourish and respect nature	River promotes well-being of local people, tourists, and recreational users River basin is an example of ecologically and socio-culturally sustainable region	River basin provides ideal circumstances for all living beings and for doing in the nature
Economy	Well-functioning forestry	Local people and landowners consider river basin as their home instead of resource storage	Vivid economy led by forestry and related processing activities

Climate change mitigation was only marginally covered in the visions identified in the future workshop, but the questionnaire results revealed its importance for the respondents. The response option that climate change mitigation should be significantly increased through the land use of the region was chosen by 52% of the respondents, 27% considered that the region should live up to national climate policy objectives, while 15% considered that climate change mitigation should guide all land use in the region. However, respondents did not consider climate change adaptation as a key target.

The discussions in the future workshop revealed that economic development was considered important, especially that based on forestry, innovative agriculture, fishing tourism, nature-based tourism, and wind and solar power production in the future. However, it was recognized that tourism-based economic development could ensure mutual benefits for people and nature only at the local level while increasing carbon emissions at global level produced by e.g., flight traffic of fishing tourists. Furthermore, forestry was considered an important source of income, but logging was considered to present a threat to water quality, biodiversity, and the climate. It was concluded that the region should not become a reservation for providing carbon sequestration and storage services and biodiversity benefits, nor should it turn to an industrialized hinterland. The hope was that economic, environmental, and socio-cultural aspects would be in balance in the region in the future.

### Nature-based solutions to achieve the visions

**Fig. 4:**
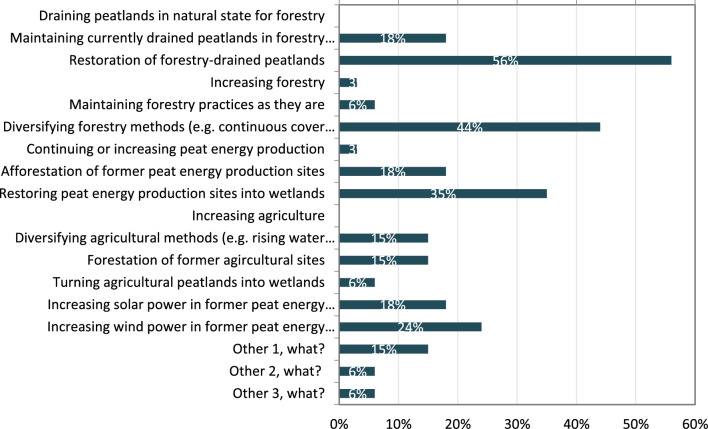
Ranking of management fixes in questionnaire (*N* = 34)

Management fixes identified in the future workshop and questionnaire were linked to ensuring that peatlands function well and would resemble their “natural state”, to hydrological manipulations in the river basin e.g., by directing water flows to peatlands and runoff fields to avoid direct flows of suspended solids and nutrients to the river and mapping the need for tailing ponds and creating them at needed locations in collaboration with landowners (Fig. 4). It was recognized that actions to increase carbon sequestration are needed, including decreasing the frequency of logging, increasing forest growth, and replacing even-age management with continuous cover forestry. The future workshop participants recognized that wide-scale restoration practices could also bring job opportunities for the local inhabitants.

An example of a successful small scale local management fix is the construction of a surface runoff wetland to Lake Juopulinjärvi in the Kiiminkijoki River basin. During the onsite visit and workshop discussions, the landowners pointed out that the new solution would probably attract waterbirds to the area and could be good for the fish as well.

Management fixes can be guided, incentivized, and directed by policy fixes to change existing behavioral practices. The questionnaire revealed interesting results regarding policy fixes. It was considered that land use decision making in the region needs major changes (41% of the responses) or that the respondents did not know enough about land use decision making to answer the question (47% of the responses). None of the questionnaire respondents considered that land use decision-making is functioning well and that no changes are needed. These results were complemented by the findings from the future workshop, where it was considered that several actors need to change their practices to enable NBS to improve water quality (Table 4).

**Table 4:** Identified changes needed in the Kiiminkijoki River basin and responsible actors based on the future workshop

Who needs to change to enable NBS	What kind of change is needed
All actors operating in the river basin	To change behavior so that it does not compromise the water quality; To perceive the river basin as a home region and not as a resource storage; To respect nature in all human activities
Local village associations; (local) ENGOs; (local) companies	To reach a common sustainable vision of the river basin
Forestry practitioners (e.g., state forestry enterprise Metsähallitus; Forest owners)	To apply more effective water protection measures in forestry
State-based organizations and legislators	To initiate new incentives and policies to enhance sustainability; To improve laws to ease the managing of waterbodies; To recognize diverse objectives and interests of various local actors
Governmental organizations monitoring forest use	To enforce water protection measures; i.e., to move from recommendations to binding responsibilities
Land use planners (e.g., municipalities) and landowners	To guide land use so that the negative impacts on water quality would be minimized; To establish a new national park in the region
Forestry interest groups, and research and advice organizations	To provide more information about alternative ways to use forests compared to current industrial forestry practices including intensive drainage; To distribute knowledge that is understandable and tailored for the purposes of local actors; To improve understanding how to ensure profitable forestry while not deteriorating quality of water
EU, national government, compensation broker companies	To establish a transparent, regulated and trustworthy carbon market through which forest owners could gain income comparable to selling wood
Fisheries districts, water management associations; actors in agriculture and forestry	To increase coordination at the river basin level; To establish position of a river basin-level wetland coordinator
Local associations; forest management associations; land-owners; Council of Oulu region;	To develop restoration plans for the sites located within the river basin
Landowners and forest owners	To change attitudes towards lands, waters, and forests from being perceived as resources to be understood as commons
Peat energy producers and owners of the peat energy production lands	To ensure that after peat energy production has ended, the sites are restored appropriately

### Scaling nature-based solutions

Discussions at the future workshop highlighted that local promising practices could be scaled-up to the river basin level by establishing a position for a river basin-level coordinator to manage multiple water protection measures by diverse actors, and to channel support for good practices. It was noted in the workshop that there is experience of using a river basin-level coordinator at the neighboring Iijoki river basin, where the coordinator has facilitated collaboration between municipalities and key actors to enhance common understanding and to build shared vision on the desirable futures of the basin.

Examples of horizontal scaling-out in the Kiiminkjoki River basin include restoration measures and management fixes (e.g., mowing water plants, management fishing, restoring rapids), that are done by local actors and village associations involving extensive local voluntary work. Scaling-out such practices was proposed to be done by borrowing machinery needed for specific restoration activities, or by ensuring financial support covering some of the costs of the work required for management fixes.

Examples of scaling-down of NBS by policy fixes in the Kiiminkijoki River basin were discussed in the workshops. First example was the potential future carbon markets, which could seek to incentivize land use solutions that can capture carbon. In the landowner workshop, it was noted that such carbon markets could function as a new source of income in the region. However, concerns of climate change and related potential compensations on carbon sequestration remained often at a rather abstract level for participants of the workshops, especially when compared to the strong perceived need to solve challenges regarding water quality in the river. In addition, doubts were raised regarding whether the carbon markets could become profitable enough to compete with economic significance of forestry in the region. Nevertheless, forest owners considered the forestry products important resource for the local economy. Another obstacle was the uncertainty what management fixes would be the best to sequester carbon. The second example mentioned in the workshops were new guidance and support systems for forest owners to manage the forests. In the past, national forest policies provided incentives for the drainage of peatlands to increase forest growth. Currently, there is increasing emphasis on developing forest policies that integrate biodiversity values and water protection into the forest management decisions (e.g., forest certifications).

In the future workshop, participants identified an extensive list of actors that should change their practices (Table 4) to enable NBS by organizational changes (i.e., scaling-in).

Scaling-deep, i.e., societal value and cultural changes, often take place in long temporal horizons. The questionnaire results revealed that around half of the respondents considered that land use decision-makers relevant to the region would need to change their decision criteria, i.e., values, to enable more sustainable practices. The economy of the region has been based on forestry and peat production for generations; thus, NBS may be seen as distracting the main economic activities that are considered emblematic in the region. This hegemonic understanding might hinder willingness for cultural change.

## Discussion

### Key ambitions of NBS

**Fig. 5:**
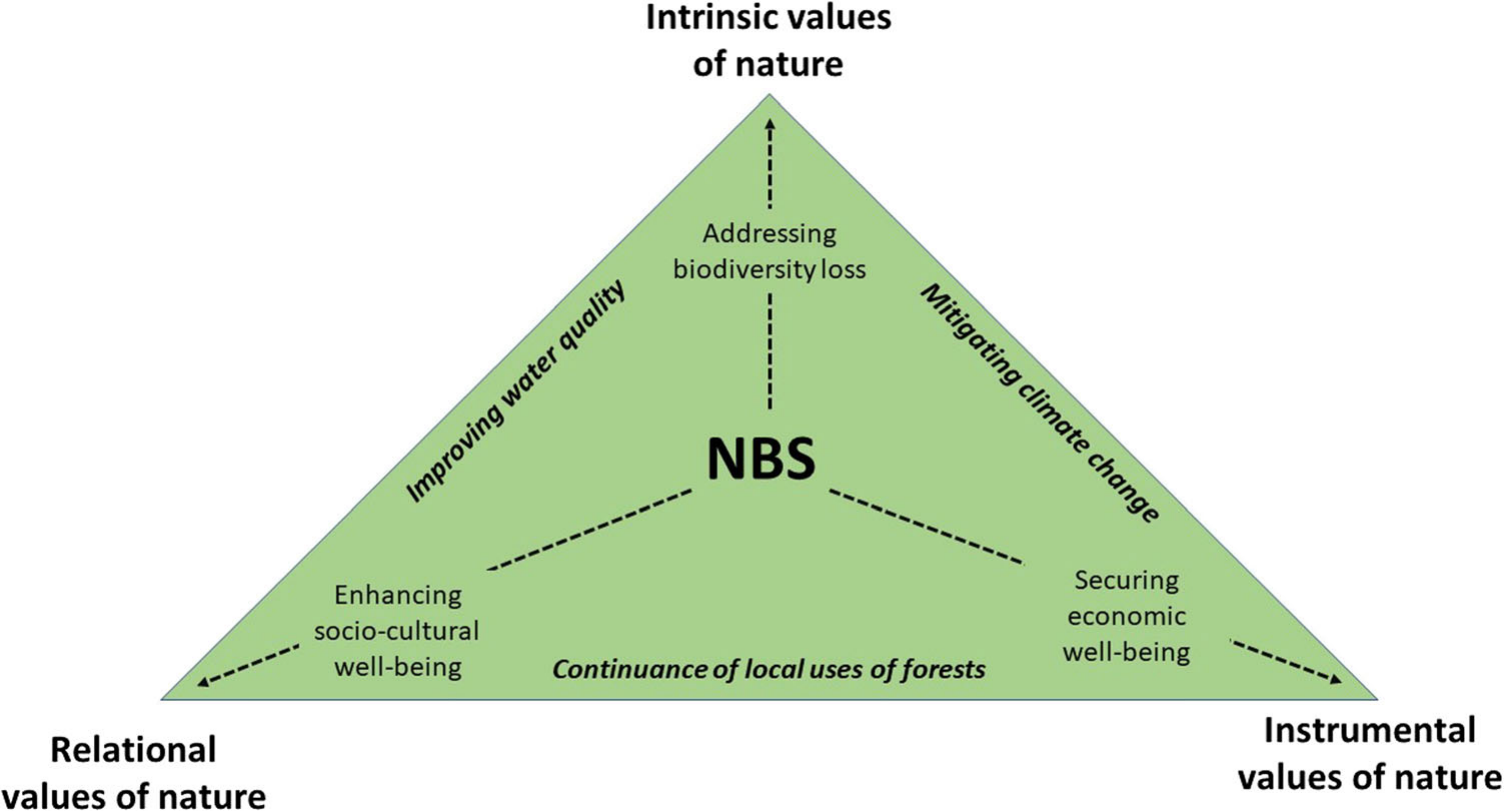
Contributions of NBS to three general value perspectives and to locally important objectives

The ability to address sustainability challenges has been considered as a key potential major contribution of NBS (Chausson et al. 2020; Seddon et al. 2020; Dumitru and Wendling 2021). However, NBS discourse has been considered to undermine ecological aspects for the favor of impacts on human wellbeing (Alva 2022). However, our results highlight that the NBS literature seems to lack the cultural aspects of sustainability. Our analysis found that cultural values related to nature are powerful motivators for many local actors to promote or oppose NBS. For instance, in the future workshop many of the participants hoped that the Kiiminkijoki River basin would not be considered simply as storage for resources (instrumental values of nature) or as totally conserved location to enhance carbon sequestration and biodiversity (intrinsic values of nature). Instead, strengthening the local nature-based livelihoods (e.g., fishing, hunting, and forestry) was considered an important objective to guide land use in the future (relational values of nature). Therefore, relational values of nature are not just curiosities, contingent articulations of cultural diversity, but instead are the manifestations of interdependent and co-existing human and nonhuman livelihood practices and hence there is a need to better recognize them in NBS to allow their thriving and potential mainstreaming. We thus support the argument that relational values can function as leverage points for policy development at the local and even the European level (Mattijssen et al. 2020). Yet, we do not propose to abandon the intrinsic and instrumental motivations of NBS. Instead, NBS should be thought to function at the intersections of instrumental, intrinsic, and relational values of nature (Fig. 5).

### Mainstreaming nature-based solutions through scaling

Implementing NBS is often slowed down by value and valuation related barriers in governance (Kabisch et al. 2016). Figure 5 highlights the interrelations between the three general value perspectives. Thus, mainstreaming NBS through the five forms of scaling also needs to address such interrelations. Occasionally, the three value perspectives are considered to be held by different actors. However, our findings indicate that same local people in the Kiiminkijoki River basin hold simultaneous, but to varying degrees, elements of all the three value perspectives. Nevertheless, collaborative governance approaches are needed to balance ways to use nature in a way that does not significantly undermine any of the three value perspectives, as also noted in the context of NBS literature (van der Jagt et al. 2017; Malekpour et al. 2021; Zingraff-Hamed et al. 2021). Changes in ways to balance the three value perspectives can start from local examples but eventually require cultural and societal change. The NBS can catalyze such change by mainstreaming change through the five forms of scaling. Yet, the cultural and societal change happens at different paces and time horizons with the five forms of scaling (Table 5). Overall, we believe that our lessons learned regarding the five forms of scaling bring the ambitions placed on NBS to address sustainability challenges a step closer to reality.

**Table 5:** Five forms of scaling and their divergent temporal horizons with examples from the case study

Form of scaling	Direction of scaling	Time horizon	Examples from the case study
Scaling-up	From site-specific good practices to policy and governance	Medium: scaling-up requires policy and governance change and pace of change depends on consensus among decision-makers	A river basin-level coordinator was established in the neighboring Iijoki River basin after a pilot project agreement between municipalities, enterprises, and regional authority (https://micropolis.fi/en/iijoki-river-agreement/)
Scaling-out	From site-specific good practices to other locations	Short–long: scaling-out depends on how fast practices are emulated in nearby areas and society writ large	Scaling-out NBS require often voluntary work by civil society actors. Scaling-out can be paced up by resourcing grassroots activities
Scaling-down	From policy to site-specific practices	Medium: depends how fast new decisions proceed in policy cycle from problem identification through policy formulation to implementation	Scaling-down carbon and ecosystem value markets requires institutional changes for example in form of compensations to forest owners. Even over a decade after Finnish voluntary forest protection program METSO’s pilot phase, ecological compensations remain at a pilot level
Scaling-in	From socio-political contexts to organizational values and practices	Medium: requires changes in internal logics of how organizations work. It may not be easy to change organizational directions overnight	In the future workshop, it was considered that a change in municipalities’ practices may be hindered by the merging of neighboring municipalities to the city of Oulu, making the administration confusing and less focused on remote rural areas
Scaling-deep	From sustainability visions to culture and society	Long-term: cultural and value changes in society happen slowly	In the case study region, plausible cultural value change from instrumental economic values of forests to simultaneous consideration of instrumental, intrinsic, and relational values is ongoing but slow. In the future workshop, it was considered that this may be achieved only by the next generation

Our case showed that site-specific promising practices could be scaled-up by governance innovation: by establishing a position for a river basin-level wetlands coordinator. Earlier, Fastenrath et al. (2020, p. 63) concluded that up-scaling needs diverse expertise and intermediaries that can *“provide platforms of ongoing exchange between the heterogenous stakeholders from public and private sectors, academia and society.”* Such intermediaries could, for example, work at the landscape level to enhance the interface between local and landscape scales and bring together various actors (see Schröter et al. 2022). Our results imply that building trust between various actors operating in the basin would be among the main challenges of a river basin-level coordinator.

Scaling-out NBS can be promoted by horizontal learning from site-specific good practices (Raymond et al. 2017). We found that the active participation of citizens and village associations in practical environmental work show a high degree of motivation to act for nature. It would be key for policy fixes to support, supply, and enable such voluntary work, and to strengthen the reciprocal stewardship and spontaneous connections between people and their environments (c.f. Diver et al. 2019; Ojeda et al. 2022). Our case showed that citizens are often active and feel responsibility especially towards water quality in the Kiiminkijoki River. Policy fixes could provide opportunities for environmental management projects by village associations and citizens including, for example, concrete actions such as planting trees and constructing bottom dams. The key challenge for scaling-out is to support grassroots level environmental management practices by supporting meaningful actions with nature.

Further research is needed to better connect locality-led bottom-up and policy-led top-down approaches to mainstream NBS (Schröter et al. 2022). Scaling-down NBS by policy can be based, for example, on forest certifications and advice given to forest owners by state-based advisory organizations. In our case study, scaling-down NBS linked to potential extension of carbon markets, for example. However, carbon markets would change the whole logic by which forest owners relate to their forests, from aiming at producing sellable cubic meters of wood to producing carbon sequestration and storage to be sold. Thus, a key obstacle for scaling-down climate change mitigation solutions was incomplete and uncertain information on the preferable and best practices. This links to ideas of Odongo et al. (2022) that NBS should be “no regret” solutions that devise strategies to maximize positive outcomes and minimize negative outcomes. Therefore, reduced uncertainty in knowledge about carbon sequestration through land use could also help forest owners decide about implementing new land use solutions without fear of regret after adopting the new practices. However, only reducing scientific understanding does not necessarily imply reduced transparency and understandability of the scientific carbon sequestration models for lay people. Yet, understandability is key when forest owners are making decisions about the use of their forests.

We demonstrated above that scaling-in the change in socio-political contexts to organizational practices needs not only target municipalities (e.g., Hawxwell et al. 2019) but various set of actors (Table 4). While many challenges for mainstreaming NBS are organization-specific, an idea put forward in the future workshop was to increase consideration of the region as a common place for which all actors are responsible, for example, by providing collaborative spaces for actors to come together and develop visions for the future.

Scaling-deep requires transformative change, including changes in values (Palomo et al. 2021), institutions and cultures (Davies and Lafortezza 2019), and understanding how the implementation of NBS initiate new synergies and trade-offs between benefits which various actors gain from nature (Raymond et al. 2017). Our findings propose that scaling-deep necessitates changing the dominant ideas of considering nature simply as natural resources or something to be fully conserved. Instead, desirable value positions were considered as something that would enable livelihoods but in a way that ensures ecological sustainability and enhances local possibilities to relate to nature (c.f. Fig. 5).

## Conclusion

Based on the results and analysis presented above, we make three key conclusions on NBS and cultural and societal change. Firstly, the relevance of NBS can be enhanced for local communities by seeking to link them with local nature-based activities. We have showed that NBS will be seriously crippled without recognition of the relational value dimension embedded in local cultures. NBS are difficult to implement without building dynamic and mutually beneficial relationships between people and nature that situate within the socio-cultural context and respect local practices and values. In the Kiiminkijoki River case, the wish for improved water quality can be seen as a key motivation for local actors to support NBS. Yet, local communities are not homogenous. For example, forest and landowners have also economic motivations linked to their forests and lands. Where these aspects are compromised, compensations, incentives, or other market-based governance instruments, such as carbon trading or nature value trading (see Neuteleers 2022), can smoothen the change in ways to use the land and water areas. However, such new economic governance instruments possibly create less economic revenues than primary production in forests and peatlands. Thus, new locally rooted innovative ideas are also needed to generate transformations towards sustainability, not only in economic but also in environmental and socio-cultural terms.

Second, our results highlight that considering the three value positions incompatible with each other is often not constructive, because they together constitute the necessary value-basis for viable cultures and societies and peoples’ relations to nature. For example, accusing forest owners of focusing solely on economic revenues is too simplistic. Another fallacy is to consider that local communities have some kind of special values and practices that can be captured only by the concept of relational values. Instead, we propose that at best, the instrumental, intrinsic, and relational value positions can be enhanced simultaneously and balanced with each other by NBS. Our results imply that halting biodiversity loss, mitigating climate change, improving water quality, and supporting the continuance of local land and water use practices can be addressed simultaneously in some situations. For example, the construction of new wetlands combined with nature value and carbon trading can address the environmental and social objectives.

Thirdly, a key strength of local NBS is that they fit to local socio-cultural and environmental contexts, but they also can be mainstreamed in society through the five forms of scaling. Our geographical focus on a river basin proved to be constructive by forming important boundaries of a region where scaling of NBS can be done. While our findings on NBS and their mainstreaming through the five forms of scaling derive from one river basin, they can inform changes in (inter)national policies of river basin management, and also site-specific practices in other river basins. Finally, while the cultural and societal value change is difficult to reach or even to document and holistic sustainability transformation remains a distant but well-grounded normative call, our results provide ideas how NBS can be linked to such change and how obstacles can be overcome to enable a transformative change for sustainability.
